# Conservation biology of threatened Mediterranean chasmophytes: The case of *Asperula naufraga* endemic to Zakynthos island (Ionian islands, Greece)

**DOI:** 10.1371/journal.pone.0246706

**Published:** 2021-02-19

**Authors:** Anna-Thalassini Valli, Vassiliki Lila Koumandou, Gregoris Iatrou, Marios Andreou, Vasileios Papasotiropoulos, Panayiotis Trigas

**Affiliations:** 1 Laboratory of Systematic Botany, Department of Crop Science, School of Plant Sciences, Agricultural University of Athens, Athens, Greece; 2 Genetics Laboratory, Department of Biotechnology, School of Applied Biology & Biotechnology, Agricultural University of Athens, Athens, Greece; 3 Division of Plant Biology, Laboratory of Botany, Department of Biology, University of Patras, Patras, Greece; 4 Nature Conservation Unit, Frederick University, Nicosia, Cyprus; 5 Department of Agriculture, University of Patras, Amaliada, Greece; Brigham Young University, UNITED STATES

## Abstract

*Asperula naufraga* is a rare and threatened obligate chasmophyte, endemic to Zakynthos island (Ionian islands, Greece). In this study, we provide a combined approach (including monitoring of demographic and reproductive parameters and study of genetic diversity) to assess the current conservation status of the species and to estimate its future extinction risk. The five subpopulations of *A*. *naufraga* were monitored for five years (2014–2018). Population size markedly fluctuated between 68–130 mature individuals during the monitoring period. The extent of occurrence (EOO) was estimated at 28.7 km^2^ and the area of occupancy (AOO) was 8 km^2^. Stage-structure recordings were similar for all subpopulations, characterized by high proportions of adult and senescent individuals, following a common pattern, which has been observed in other cliff-dwelling plants. Preliminary genetic analysis with SSRs markers revealed low heterozygosity within subpopulations and significant departure from H-W equilibrium, which combined with small population size suggest increased threat of genetic diversity loss. Our results indicate that the species should be placed in the Critically Endangered (CR) IUCN threat category, while according to Population Viability Analysis results its extinction risk increases to 47.8% in the next 50 years. The small population size combined with large fluctuations in its size, low recruitment and low genetic diversity, indicate the need of undertaking effective *in situ* and *ex situ* conservation measures.

## Introduction

The Mediterranean Basin constitutes the second largest hotspot of plant diversity and endemism at a global scale [1]. The diverse topographies of the Mediterranean landscape have largely been shaped by tectonic activity that has formed extensive mountain systems and impressive geological reliefs. Large cliff systems are scattered almost throughout the Mediterranean, hosting a specialized chasmophytic flora. Obligate chasmophytes are exclusively cliff-dwelling specialists adapted to withstand the extreme conditions imposed by a vertical habitat [2, 3], and constitute a large proportion of the Mediterranean endemic plants [4]. Obligate chasmophytes constitute about 7% (476 species and subspecies) of the Greek vascular flora and 21% of the Greek endemic flora, with about 85% of them being endemic to Greece and/or having a restricted distribution range [5, 6].

Habitat specialization to the harsh conditions of cliff surface often leads to low dispersal ability and rarity [7, 8]. However, absence of grazing and low level of inter-specific competition are the rewards for a life at the extreme. Reduced dispersal abilities combined with low habitat availability/connectivity may cause population to decline and a reduction in geographical range for several chasmophytes under future climate change. Although cliff-dwelling plants are especially threatened by climate change [9], they have been largely overlooked by conservation scientists. Human-caused habitat loss has probably been the most common form of biodiversity loss for millennia, and plants growing in the most affected habitats e.g., coastal sand-dunes, pastures, wetlands, etc., have received increased conservation efforts and resources. Climate change has altered the conservation agenda in the last decades, but chasmophytes remain neglected for an additional reason; their habitats are usually inaccessible, making collection of field data an arduous and potentially dangerous task. Hundreds of threatened obligate chasmophytes are growing in the cliff systems of the Mediterranean Basin, and only few of them have been thoroughly studied [e.g., 8, 10–15]. Understanding population dynamics and genetic diversity of chasmophytes, as well as the factors shaping them and their potential implications, are key issues for their effective conservation.

The advantages of multidimensional approaches regarding the biology and ecology of threatened plants towards the implementation of effective conservation management planning have been repeatedly highlighted [e.g. 16, 17]. However, most plant conservation studies mainly focus on either a biological/demographic or genetic approach [18, 19]. Both approaches are rarely combined and are even more rarely accompanied by monitoring data [8]; the latter is irreplaceable in identifying declining populations at risk of extinction and assessing the effectiveness of specific conservation management plans [19, 20].

Understanding genetic structure and variability of plants facilitates investigation of their biological characteristics, evolutionary history, and adaptive potential [21]. Endemic species, in particular, often having small and fragmented populations, are prone to inbreeding and loss of genetic diversity [22]. Preserving genetic diversity is essential in plant conservation planning, since intra- and inter-population genetic variability is essential for maintaining a high level of adaptive potential to cope with new selection pressures caused by environmental change [23]. Moreover, genetic studies can improve the efficiency of conservation activities by identifying populations with low and/or high genetic diversity and by providing information regarding the extent of gene flow among fragmented populations [24, 25].

The west coasts of the Ionian Islands (Greece) have some of the largest and most impressive cliff systems in the Mediterranean, hosting a rich chasmophytic flora. *Asperula naufraga* Ehrend. & Guterm. is a perennial chasmophyte, forming loose cushions [26]. It is a local endemic of Zakynthos Island, growing on calcareous cliffs and crevices in the west coast cliff systems of the island, at 2–265 m a.s.l., and has been classified as Endangered (EN) [27]. It is a member of *Asperula* sect. *Cynanchicae* (DC.) Boiss. and had been provisionally included in the informal *A*. ser. *Palaeomediterraneae* [26, 28]. The taxa of this group have a scattered distribution from the islands of the Adriatic and the Ionian Sea to the southern Italian Peninsula, the Balearic Islands, and the adjacent coast of SE Spain. However, a recent phylogenetic reconstruction of *A*. sect. *Cynanchicae* revealed that this group does not represent a monophyletic unit and turns out to be highly artificial [29]. Phylogenetic relationships within *A*. sect. *Cynanchicae* are blurred because of poor species divergence and hybridization, combined with some cases of non-monophyletic species [29]. *Asperula naufraga*, however, is a well-differentiated species in morphological terms, easily distinguished from all related species within *A*. sect. *Cynanchicae* [26]. Information on its mating system is lacking. Perennial members of the tribe Rubieae are mainly outbreeders due to self-incompatibility through mechanisms of dimorphic heterostyly [30–32]. Autogamy has been documented only for *Asperula daphneola* O. Schwarz [33].

In this study, we combine demographic and genetic approaches to assess the current conservation status of *A*.*naufraga* and to estimate its future risk of extinction. More specifically, we aim to: (a) accurately define the geographical distribution of the species after exploring all potentially suitable habitats, (b) assess its population dynamics and reproductive biology, (c) estimate genetic diversity and potential gene flow within and among subpopulations, and (d) propose strategies and conservation measures for its management and maintenance.

## Materials and methods

### Definitions

The terms mature individual, population, subpopulation, population size, location, Extent of Occurrence (EOO) and Area of Occupancy (AOO) are used according to the definitions established by the International Union for the Conservation of Nature [34]. EOO is defined as the area contained within the shortest continuous imaginary boundary which can be drawn to encompass all the known, inferred or projected sites of present occurrence of a taxon, excluding cases of vagrancy. EOO can often be measured by a minimum convex polygon. AOO is defined as the area within EOO which is occupied by a taxon excluding cases of vagrancy. The measure reflects the fact that a taxon will not usually occur throughout its EOO [34]. Local EOO was calculated according to Andreou et al. [35] as the minimum area occupied by individuals of each subpopulation.

### Spatial distribution

All known locations, as well as all suitable habitats for *A*. *naufraga* (calcareous cliffs, steep rocky slopes facing the sea) were surveyed for five consecutive years (2014–2018), in order to identify its distribution range. The exploration of the west coast cliffs of Zakynthos was aided by the use of binoculars. A GPS device was used for the capture of location data in the field. Detailed mapping, the polygon of the EOO for the total population, as well as the polygons of the local EOO for each subpopulation of the species were constructed using the ArcGIS 10.5.1 (ESRI) software package. AOO was estimated as the sum of the occupied grid cells 1 × 1 km.

### Population size

For the estimation of population size (i.e. the total number of mature individuals in all subpopulations), censuses of the mature individuals during the flowering/fruiting period in all subpopulations were carried out for five consecutive years. As an individual in this study we defined a cluster of shoots, in order to exclude the possibility of counting potential clones, although there is no evidence that the species can reproduce vegetatively. The number of plants per m^2^, which gives a rough estimation of plant density [19], was estimated for each subpopulation dividing the number of mature individuals by the local EOO. In addition, for the investigation of the stage-structure distribution of the species, plants were classified into three categories: seedlings, non-reproductive plants (juveniles and non-flowering) and reproductive plants (flowering/fruiting), as suggested by Soriano et al. [9]. Annual censuses of individuals per life—stage in each subpopulation were carried out for the whole monitoring period.

### Reproductive biology

For the study of reproductive characteristics, i.e., fecundity (expressed as mean number of seeds produced per individual) [36] and Relative Reproductive Success (RRS, i.e. the total percentage of all ovules maturing into seeds), 5–10 randomly selected mature individuals per subpopulation were tagged at the beginning of the flowering season in the most easily accessible subpopulations of the species, i.e. An-Pl, An-PV and An-N (Table 1), for five consecutive years. Tagging was implemented with an indelible marker on the cliff surface close to the individual, in order to avoid plant damage. All monitoring and experimental procedures were approved by the Hellenic Ministry of Environment and Energy, Directorate of Forest Protection (approval no. MEE/DFP/125613/6014).

**Table 1:** Geographical data of *Asperula naufraga* subpopulations.

Location	Subpopulation abbreviation	longitude	latitude	Altitude (m)	Slope (°)	AOO (km^2^)
Plakaki	An-Pl	20.771043°	37.684911°	2–60	45–120	1
Faros Keriou	An-F	20.805406°	37.655566°	53–67	60–110	1
Porto Vromi	An-PV	20.631753°	37.830836°	2–265	45–90	4
Navagio	An-N	20.625887°	37.862830°	190–230	45–90	1
Sxiza	An-S	20.679972°	37.781673°	144–148	45–90	1

The number of flowering stems per individual, and the number of flowers per flowering stem and per individual were recorded during each flowering period. The number of mericarps per stem was recorded during each fruiting period (the fruit of *A*. *naufraga* is a schizocarp, consisting of two mericarps, each one containing a single seed). In order to estimate the number of sound seeds per flower and per stem, 1–3 stems were collected from tagged individuals during each fruiting period and the seeds (seeds remain attached to the fruit during the first stages of its formation) were observed with a stereoscope (ZEISS Stemi 305). RRS was calculated by dividing the actual production of sound seeds to the potential maximum seed production. Seed rain was estimated according to Andreou et al. [19] by multiplying the estimated number of seeds per individual by the number of mature individuals in each subpopulation and dividing by the local EOO of each subpopulation. The survival rate of the mature individuals (Sa) (i.e. the proportion of mature individuals that survive from one breeding season to the next) was studied by tagging 20 randomly selected mature individuals from subpopulations An-Pl, An-PV and An-N during the flowering period and checking their viability in the following flowering period, since data collected from permanently marked individuals enable more accurate and precise parameter estimation [37, 38]. Generation length (which is used as a time-scalar in the Red List as a way of accounting for differences in species’ life-histories) was calculated as (α+1/ (1-Sa)), where α is the age of first reproduction (in years) and Sa is the annual survival rate of mature individuals [39].According to our observations, the age that *A*. *naufraga* reaches its first reproduction is 3 years.

The duration of flowering (and fruiting) period of *A*. *naufraga* was monitored every 1–2 weeks over five consecutive years in all subpopulations. The association of annual climate data (mean temperature, maximum temperature, minimum temperature, and precipitation) and the duration of flowering period was examined with stepwise multiple linear regression analysis, in order to investigate the impact of climatic variables on flowering period. Meteorological data were obtained from Hellenic National Meteorological Service. Statistical analyses were performed using Statistica 8.0 software (StatSoft Inc.). Moreover, to identify possible pollinators, all insect species that were associated with *A*. *naufraga* pollination were photographed and samples were collected for further identification to the lowest possible taxonomic level.

Comparisons of data regarding reproductive biology were performed by One Way ANOVA. Differences among pairs of means were checked by Tukey’s Method using Statistica 8.0 software (StatSoft Inc.).

Population Viability Analysis (PVA) was carried out using the total number of mature individuals during the first year of monitoring (i.e. 2014) as initial abundance and the survival rate of mature individuals as survival rate, taking into consideration demographic stochasticity and without considering any density-dependent parameters. Population growth calculated as the interannual variation in the total number of mature individuals (N_t_/N_t+1_). The analysis was implemented with provision for the next 10 and 50 years, using RAMAS Ecolab v.2 software package [40].

The assessment of the conservation status of *A*. *naufraga* was made following the guidelines of IUCN Red List Categories and Criteria [41], and using Ramas Red List Professional software [39].

### Threats

The direct threats that have impacted, are impacting, or may impact the status of *A*. *naufraga*, as well as the stresses they cause to the species were recorded and classified according to IUCN [34].

### Genetic analysis

For genetic analysis, a total of 63 individuals from the five subpopulations were sampled (young shoots/stems with leaves) in March 2018. All sampled individuals were located at least 5 m apart. The number of individuals sampled depended on the size and the accessibility of each subpopulation examined (Tables 1 and 2), while at the same time plant tissue collection was not destructive for the plants. Plant tissues were dried in silica gel with color marker and then transferred to the laboratory. 50–100 mg of plant tissue were ground in liquid nitrogen and total genomic DNA was extracted from every individual using the NucleoSpin Plant II kit (Macherey-Nagel) according to the manufacturer’s instructions. Quantification and quality assessment of the purified DNA were performed spectrophotometrically using a Nanodrop ND 1000 Spectrophotometer. DNA samples were stored at −20°C until further analysis.

**Table 2:** Number of mature individuals (subpopulation size), seedlings, non-reproductive individuals and dead individuals, local EOO and plants per m^2^ for each subpopulation per year.

Subpopulation	Year	Subpop. Size	Seedlings	Non reproductive	Dead individuals	Plants/m^2^	L. EOO (m^2^)
**An-Pl**	**2014**	28	0	8	0	0.003	9100.9
	**2015**	22	5	5	2	0.0024	9100.9
	**2016**	20	3	4	0	0.0022	9100.9
	**2017**	25	8	18	0	0.0027	9100.9
	**2018**	28	6	22	2	0.003	9100.9
**An-F**	**2014**	30	2	10	0	0.031	961.6
	**2015**	28	0	6	0	0.029	961.6
	**2016**	12	0	9	0	0.012	930
	**2017**	14	0	5	0	0.015	930
	**2018**	25	5	12	0	0.026	930
**An-PV**	**2014**	46	3	1	0	0.001	44642
	**2015**	37	3	2	0	<0.001	44642
	**2016**	25	0	7	1	<0.001	44642
	**2017**	28	2	5	2	<0.001	44642
	**2018**	33	2	7	2	<0.001	44642
**An-N**	**2014**	20	2	0	0	0.0012	16713
	**2015**	13	0	3	0	<0.001	14500
	**2016**	5	0	2	0	<0.001	11100
	**2017**	10	0	5	0	<0.001	11100
	**2018**	19	1	9	2	0.001	14400
**An-S**	**2014**	6	1	2	0	0.47	12.9
	**2015**	9	0	0	0	0.7	12.9
	**2016**	6	0	0	3	0.47	12.9
	**2017**	2	2	3	0	0.16	12.9
	**2018**	5	0	3	0	0.39	12.9
**TOTAL**	**2014**	**130**	**8**	**21**	**0**		**71430.4**
	**2015**	**109**	**8**	**16**	**2**		**69217.4**
	**2016**	**68**	**3**	**22**	**4**		**65785.8**
	**2017**	**79**	**12**	**36**	**2**		**65785.8**
	**2018**	**110**	**14**	**53**	**6**		**69085.8**

Subpopulation abbreviations as in Table 1.

After preliminary testing of five nuclear microsatellite loci already developed for the closely related *Asperula crassifolia* L. [42], three were used for screening genetic diversity of *A*. *naufraga* subpopulations, as a preliminary attempt to collect genetic information about a species which is extremely rare and threatened. PCR amplifications were carried out in a final volume of 25 μL reaction mixture containing: 50 ng genomic DNA, 1 X Taq buffer (Kapa Biosystems), 0.3 mM dNTP Mix (Kapa Biosystems), 0.3 pmol/μL forward primer, 0.3 pmol/μL reverse primer, 1 U HiFi HotStart DNA Polymerase (Kapa Biosystems). Reactions were carried out in a thermal cycler (Bio-Rad Laboratories Inc.) under the following conditions: an initial denaturation step at 95°C for 3 min., followed by 35cycles of 20 s denaturation at 98°C, annealing at 60–62°C (depending on the primer pair: 62°C for GA_1, 60°C for GA_50A and GA_30C) for 15 s, extension at 72°C for 30 s. The PCR program was completed by a final extension step at 72°C for 1 min. Negative controls without DNA were used to exclude (cross) contamination in the PCR reaction. The PCR products were separated by electrophoresis on a 3% agarose gel (BDH Laboratory Supplies) in 0.5 X TBE buffer, stained with Midori Green (Nippon Genetics) and visualized using Alpha Imager-Mini (Alpha Innotech). Molecular weights were estimated by comparison with a 20-bp DNA ladder (TaKaRa Bio Inc).

The population genetic software GenAlEx ver. 6.502 [43] was used for the estimation of genetic diversity parameters: number of alleles per locus (*N*_a_), number of effective alleles per locus (*N*_e_), unbiased expected heterozygosity (u*H*e), observed heterozygosity (*H*_o_), percentage of polymorphic loci (*P*) and Shannon’s index of diversity (*I*). GenAlEx was also used to perform Chi-Square Tests for Hardy-Weinberg Equilibrium for the subpopulations studied.

The Polymorphism Information Content (PIC) for each SSR locus was calculated from allele frequencies [44, 45] with the formula implemented in the program CERVUS v.3.07 (Field Genetic Ltd, London, UK). PIC value provides an estimate of discriminatory power of the markers considering not only the number of alleles per locus but also their relative frequencies in the population studied [46].

Unbiased genetic distances (u*D*) between subpopulations were estimated according to Nei [46]. Fixation Index-Inbreeding coefficient (*F*) on a per locus basis and Wright’s *F*-statistics [47] including, *F*_IS_, *F*_IT_ and *F*_ST_ were calculated using GenALEx. The estimation of gene flow (*Nm*), calculated as the number of migrants entering a subpopulation in each generation, was made according to Wright [48]. The presence of null alleles was checked by Micro-Checker 2.2.3 [49]. In order to assess the effect of null alleles and to correct the bias induced by their presence on the *F*_ST_ estimation, we used the Excluding Null Alleles (ENA) method as implemented in the program FreeNA [50], using 10,000 bootstrap replications. To infer population structure and assign individuals to populations, we used STRUCTURE version 2.3.1 [51]. No prior knowledge of the subpopulations was included in the analyzed dataset. To determine the optimal number of clusters (K), twenty runs of STRUCTURE were performed by setting the number of clusters (K) from 1 to 5 (equal to the number of subpopulations), with a length of the burn-in period of 100,000 steps followed by 50,000 Monte Carlo Markov chain (MCMC) replicates, assuming an admixture model and correlated allele frequencies.

The ΔΚ method based on the criteria proposed by Evanno et al. [52] and the obtained posterior probability values [51] were used to determine the optimal number of clusters (Κ) in Structure Harvester [53]. To obtain a consensus barplot for the 20 interactions performed the CLUMPAK [54] platform was used.

The distribution of genetic variance within and among subpopulations was investigated by Analysis of Molecular Variance (AMOVA), based on the hierarchical variance of gene frequencies. The test of significance for the AMOVA was carried out on 9,999 permutations of the data. Principal Coordinate Analysis (PCoA) based on Nei’s genetic distances and on the pairwise *F*_ST_ distance matrix calculated with FreeNA and corrected with EM algorithm for null alleles, was carried out in GenAIEx software.

Effective population size (*Ne*) (i.e. the size of the ideal population with the same rate of genetic drift as in the actual population being considered [48] was estimated by multiplying the initial abundance (*Nc*) (i.e. census number of mature individuals during the year 2014) by 0.4 and 0.1, representing a common range of *Ne*/*Nc* ratios as suggested by Garner et al. [55].

## Results

### Geographical distribution

**Fig 1:**
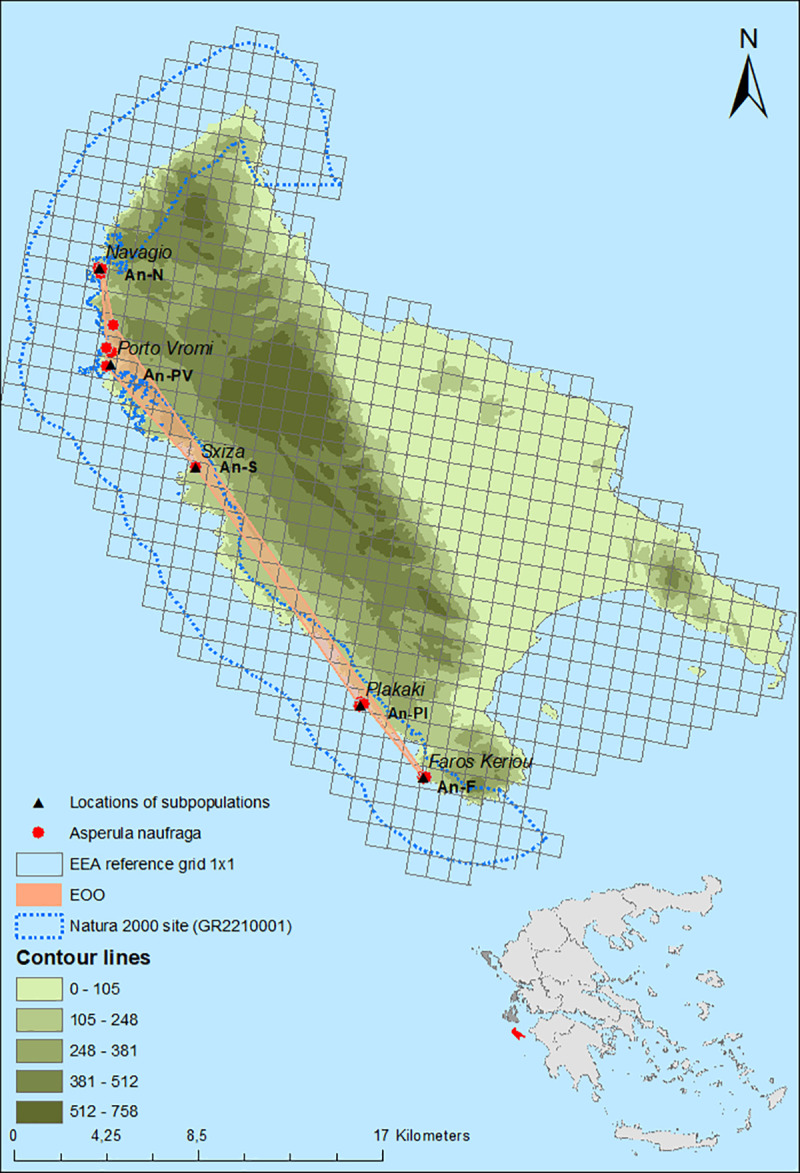
Geographical distribution of *Asperula naufraga* subpopulations (red dots) in Zakynthos Island, the estimated Extent of Occurrence (EOO), and Area of Occupancy (AOO) based on the European Environment Agency (EEA) reference grid 1 × 1 km. The data were obtained from a GPS device during field work and the map was constructed using the ArcGIS 10.5.1 (ESRI) software package.

The current distribution range of *A*. *naufraga* is shown in Fig 1. Its population consists of five subpopulations, namely Faros Keriou (An-F), Plakaki (An-Pl), Sxiza (An-S), Porto Vromi (An-PV), and Navagio (An-N), arranged along the steep western coasts of Zakynthos (Fig 1, Table 1), while the species was recorded almost at the sea level (2 m a.s.l.) for the first time. All subpopulations are included within the Site of Community Importance “Dytikes kai Voreioanatolikes aktes Zakynthou” (GR2210001) of the Natura 2000 network of protected areas. Subpopulation An-Pl was discovered during this study. According to Constantinidis & Kamari [27] there is an additional subpopulation in Marathias area (southwest Zakynthos, near Faros Keriou). Despite repeated visits to this area, however, the presence of *A*. *naufraga* at this location was not confirmed. According to population monitoring results the EOO of *A*. *naufraga* is 28.7 km^2^, the AOO based on 1 × 1 km grid is 8 km^2^ and the local EOO is 0.7 km^2^ (Fig 1 and Table 1).

### Population size

All subpopulations of *A*. *naufraga* are especially small, including 2–46 mature individuals (Table 2). Census population size (i.e. total number of mature individuals in all subpopulations), as well as the size of each subpopulation exhibited considerable annual fluctuations over the monitoring period (Table 2). The same fluctuation pattern was shared by all subpopulations, with highest number of individuals counted in 2014, then declining reaching minimum values in 2016, followed by a continuous increase until 2018. Highest values of plant density were recorded in subpopulation An-S, the subpopulation with the smallest local EOO. Stage-structure recordings (i.e. number of seedlings, non-reproductive and mature individuals per subpopulation) revealed that all subpopulations were dominated by mature individuals (60.7–82%), non-reproductive individuals represented 12–32.1%, while percentage of seedlings was smaller (5–10.2%) (Table 2). During five years of monitoring, a total number of 45 seedlings and 14 dead individuals were recorded. The sum of annual reduction of subpopulations size, however, is higher than the recorded number of dead individuals, indicating that individual loss is considerably higher than the recorded and the species is probably a short-lived perennial.

### Reproductive biology

The mean values of the reproductive characteristics of the An-Pl, An-N and An-PV subpopulations of *A*. *naufraga* are shown in Table 3. The mean number of flowers per stem and per individual range between 11.71±0.95–24.87±3.17 and 221.32–345.69, respectively. The mean number of fruits per individual ranges from 64.18 to 127.9. The number of sound seeds per fruit ranges between 0.58±0.06–0.8±0.07. The mean number of seeds per individual ranges from 89.2 to 287.5. The survival rate of mature individuals ranged from 65% in 2016 to 95% in 2018. Fecundity was highest in 2016, the year with the smallest population size and the lowest survival rate. The mean values of RRS per year were low to moderate (28.9%–40%). Pearson’s correlation coefficient revealed a significant positive correlation between RRS and the number of flowers per flowering stem (r = 0.319, p<0.05), as well as the number of seeds per fruiting stem (r = 0.53, p<0.05) (Table 3). Mean generation length of *A*. *naufraga* was estimated to seven years.

**Table 3:** Characteristics of reproductive biology and fecundity (expressed as mean number of seeds produced per individual) of *A*. *naufraga* in subpopulations An-Pl, An-PV and An-N during five consecutive years (2014–2018).

	2014	n	2015	n	2016	n	2017	n	2018	n
Stems/individual ± SE	28.3±3.9	20	13.5±1.6	20	22.05±2.9	20	18.8±2.1	20	23.5±3	20
Flowering/Fruiting stems per individual ± SE (St)	18.9±3.67	20	9.65±1.49	20	13.9±3.01	20	12.5±2.3	20	16.95±2.55	20
Mean number of flowers per stem (Fl)	11.71±0.95	40	21.55±3.03	40	24.87±3.47	40	20.94±1.75	40	19.78±3.7	40
Mean number of flowers per individual (F = Fl × St)	221.32		207.96		345.69		261.75		335.27	
Mean number of fruits per flower	0.29±0.06	40	0.34±0.06	40	0.37±0.07	40	0.33±0.07	40	0.3±0.04	40
Mean number of Fruits per individual (F × Fl)	64.18		70.71		127.9		86.4		100.58	
Sound Seeds per fruit ± SE	0.58±0.06	40	0.76±0.16	40	0.8±0.07	40	0.65±0.09	40	0.6±0.05	40
Mean number of seeds per stem (S)	7.26±1.23	40	9.24±2.79	40	20.68±3.64	40	13.04±1.66	40	11.47±2.28	40
Fecundity (mean number of seeds per individual) (S × St)	137.2		89.2		287.5		163		194.4	
Seed rain (seeds/m^2^)	0.00077		0.00078		0.00057		0.00058		0.00069	
Survival of mature individuals (%) (Sa)			85%	20	65%	20	90%	20	95%	20
Relative Reproductive Success (%)	**28.96±2.92**		**34.06±1.99**		**40±3.45**		**32.3±4.43**		**31.14±3.66**	

n: sample size (i.e. number of randomly selected mature individuals/ number of stems from tagged individuals).

**Fig 2:**
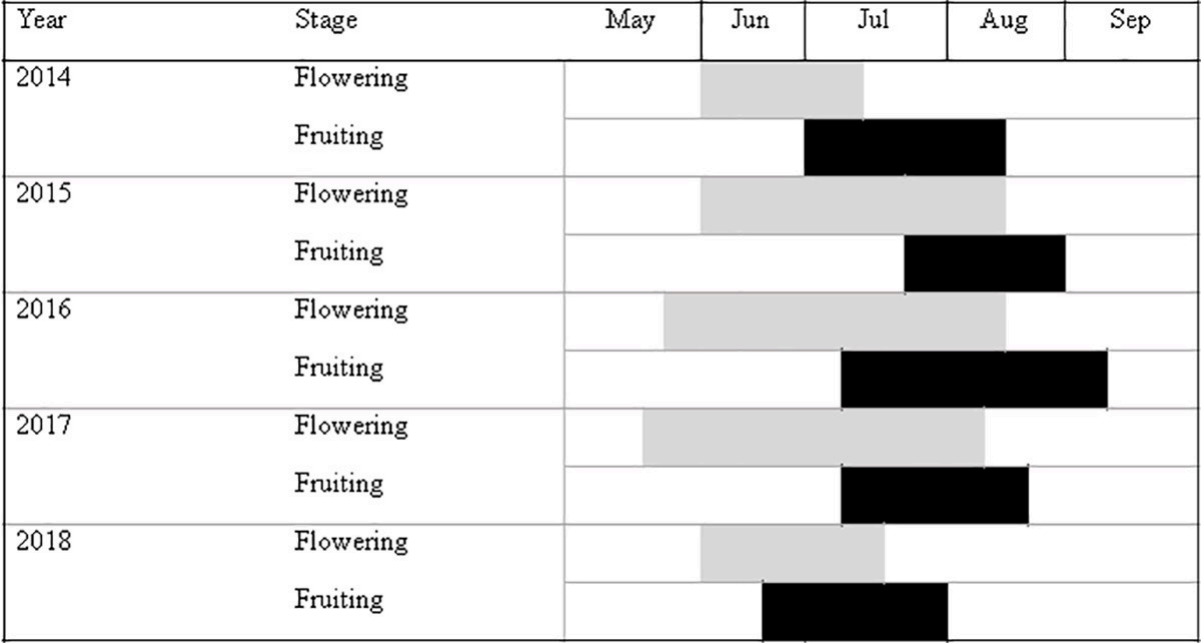
Flowering and fruiting period of *Asperula naufraga* during five consecutive years (2014–2018).

Flowering and fruiting periods of *A*. *naufraga* exhibited considerable annual fluctuations (Fig 2). Flowering period lasts 59 days on average (early June–late July), followed by fruiting period, which lasts for 42.25 days on average (early to mid-July–late August). The duration of flowering period is strongly affected by annual fluctuations in temperature and precipitation (R^2^ = 0.79). Specifically, flowering period is significantly elongated by higher mean annual temperatures (b* = 7.347, p<0.001). Contrariwise, higher minimum temperatures (b* = -6.777, p<0.001), higher maximum temperatures (b* = -5.277, p<0.001) and increased precipitation (mm) (b* = -2.496, p<0.001) lead to shortened flowering period. In addition, the duration of the flowering period seems to have a positive effect on RRS (b* = 0.335, p<0.001).

Two different insect species were repeatedly recorded on the flowers of *A*. *naufraga*, during flowering period. *Coptocephala rubicunda* subsp. *rubicunda* (Chrysomelidae, Coleoptera) is considered to be polinophagous (feeding on pollen) [56], hence the transmission of pollen between individuals of *A*. *naufraga* could be expected. The other species (*Chrysops* sp., Tabanidae, Diptera) belongs to a genus of blood-sucking insects, although it is possible that adults feeding on flower nectar may provide pollination services [57].

### Genetic diversity

Twelve alleles in total were revealed from the three amplified SSR loci in 63 individuals of *A*. *naufraga*. Since the average number of amplified alleles per locus was four (S1 Table), all microsatellite markers were retained for further analysis. The allelic size ranged from 150 to 220 base pairs. More specifically, allele size for the GA_1 locus ranged between 150–175, for GA-50A between 165–200 and for GA_30C between 190–220 base pairs. PIC value for each SSR marker was 0.452 for the GA_50A locus, 0.629 for GA_30C and 0.667 for GA_1.

The number of alleles per locus and subpopulation (*N*_a_) ranged from 2.00 for An-S to 3.67 for An-Pl and An-PV, respectively. The number of effective alleles (*N*_e_) per locus ranged from 1.71 (An-S) to 2.86 (An-PV). The Chi-Square Tests performed to test for Hardy-Weinberg Equilibrium, revealed that except for loci GA-50 and GA-1 in An-S and GA_30 in An-Pl, in all other cases the subpopulations deviated significantly from Hardy-Weinberg equilibrium.

Null alleles were detected in all subpopulations by both Micro-checker, and FreeNA. The average frequency of null alleles for each SSR marker in all subpopulations studied was 0.06 for GA_1, 0.20 for GA_50A and 0.25 for GA_30C. The only cases where null alleles were not detected were in subpopulations An-N and An-S for the GA_1 marker.

Regarding diversity parameters, the mean unbiased expected heterozygosity (or else gene diversity) of subpopulations was moderate (u*H*_e_ = 0.532); subpopulation An-S, which is the smallest subpopulation of the species, showed the lowest value (u*H*_e_ = 0.474) whereas the largest subpopulation (An-PV) exhibited the highest (u*H*_e_ = 0.661).The mean observed heterozygosity within the subpopulations of *A*. *naufraga* was relatively low (*H*_o_ = 0.278). Subpopulation An-F exhibited the lowest value (*H*_o_ = 0.178), whereas subpopulation An-N the highest (*H*_o_ = 0.359) (Table 4).

**Table 4:** Summary of genetic variability of *Asperula naufraga*.

	N	Na ± SE	Ne ± SE	P	Ho ± SE	uHe ± SE	I ± SE	F± SE
**An-Pl**	13	3.67±0.33	2.05±0.41	100	0.333±0.092	0.489±0.112	0.855±0.177	0.302±0.116
**An-F**	15	2.67±0.33	2.02±0.46	100	0.178±0.178	0.509±0.135	0.819±0.214	0.706±0.294
**An-PV**	17	3.67±0.33	2.86±0.31	100	0.255±0.071	0.661±0.045	1.148±0.112	0.614±0.09
**An-N**	13	2.33±0.33	2.08±0.24	100	0.359±0.200	0.527±0.055	0.767±0.119	0.316±0.41
**An-S**	5	2.00±0.00	1.71±0.15	100	0.267±0.133	0.474±0.059	0.615±0.058	0.306±0.367

Abbreviations as follows: **N**, sample size; **Na**, number of different alleles per locus/population; **Ne**, effective number of alleles per locus; **P**, Proportion of polymorphic loci (at least two alleles per locus); **Ho**, observed heterozygosity; **uHe**, unbiased expected heterozygosity; **I**, Shannon’s information index; **F**, Fixation index for codominant data.

The Fixation Index (*F*) on a per locus basis, representing reduction in heterozygosity, ranged from 0.302 (An-Pl) to 0.706 (An-F) (average *F* = 0.449), indicating in most cases inbreeding or undetected null alleles. *F*-statistic also indicates high inbreeding level (average *F*_IS_ = 0.457, p = 0.0001) as *F*_IS_ measures the reduction in heterozygosity, due to non-random mating within each subpopulation, and high departure of genotype frequencies from Hardy–Weinberg expectations (shortage of heterozygotes) relative to the entire population (average *F*_IT_ = 0.583, p = 0.0001) (Table 4). Shannon’s information index (*I*) varied from 0.615 to 1.148 for An-S and An-PV respectively (Table 4). Moreover, average gene flow (*Nm*) among the *A*. *naufraga* subpopulations was 1.282 individuals per generation.

Nei’s genetic distances were estimated to explore genetic differentiation levels between *A*. *naufraga* subpopulations. High pairwise genetic distances were observed between An-N and An-Pl (0.759), An-N and An-S (0.643), as well as between An-N and An-F (0.614). Low pairwise genetic distance was observed between An-Pl and An-F (0.124) (S2 Table). Likewise, the same pattern was observed based on the *F*_ST_ values calculated either with or without the ENA correction for null alleles (S2 Table).

The analysis of molecular variance (AMOVA) indicated higher within subpopulation variation which accounted for 79% of the total variation, while variation among subpopulations accounted for 21% of the total variation. The global *F*_ST_ value either calculated with (0.157) or without (0.164) the ENA correction for null alleles, indicates moderate genetic differentiation among subpopulations.

**Fig 3:**
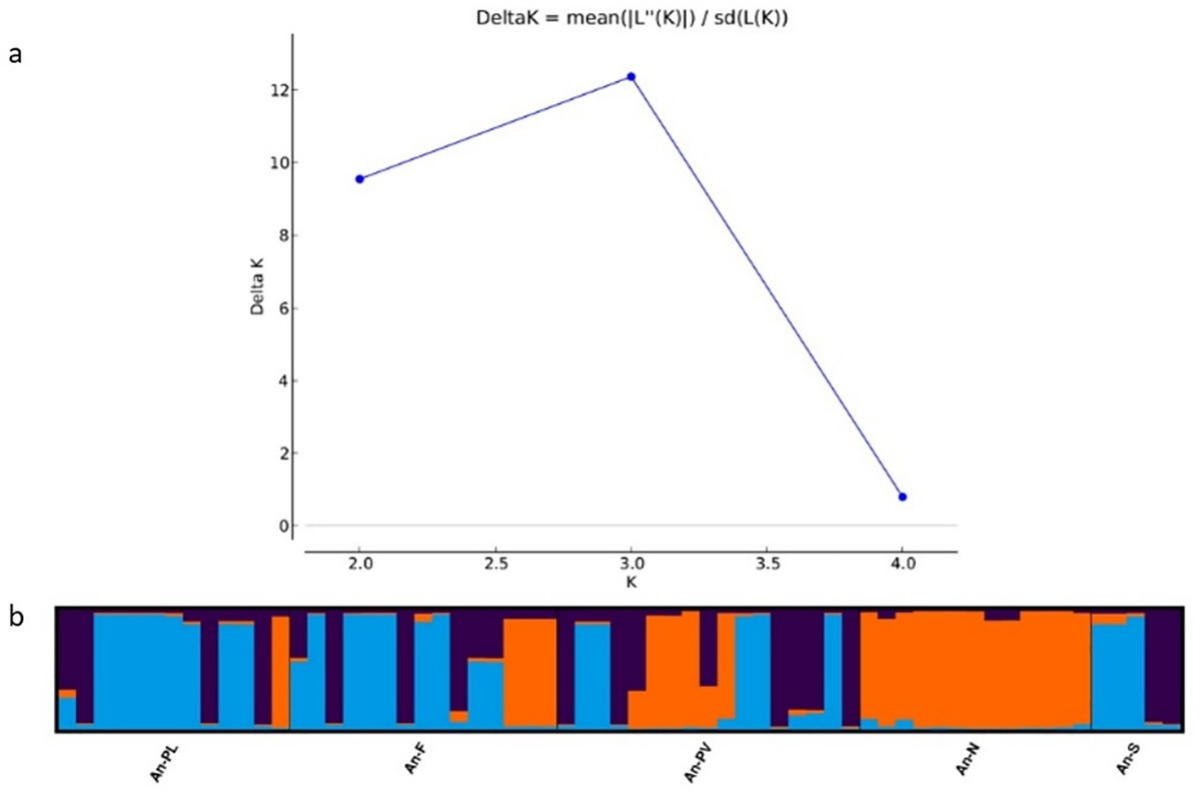
a) Delta K values for 1 to 5 genetic clusters for *Asperula naufraga* subpopulations. Delta K was calculated according to Evanno et al. [52]. b) STRUCTURE analysis barplots for K = 3 (admixture model) showing the genetic structure of the different *A*. *naufraga* subpopulations. Each individual is represented by a vertical column that reflects the individual assignment probability (*q*-value) to the respective cluster. Same color in different individuals indicates that they belong to the same cluster. STRUCTURE major mode was supported by 20 out of 20 replication runs.

**Fig 4:**
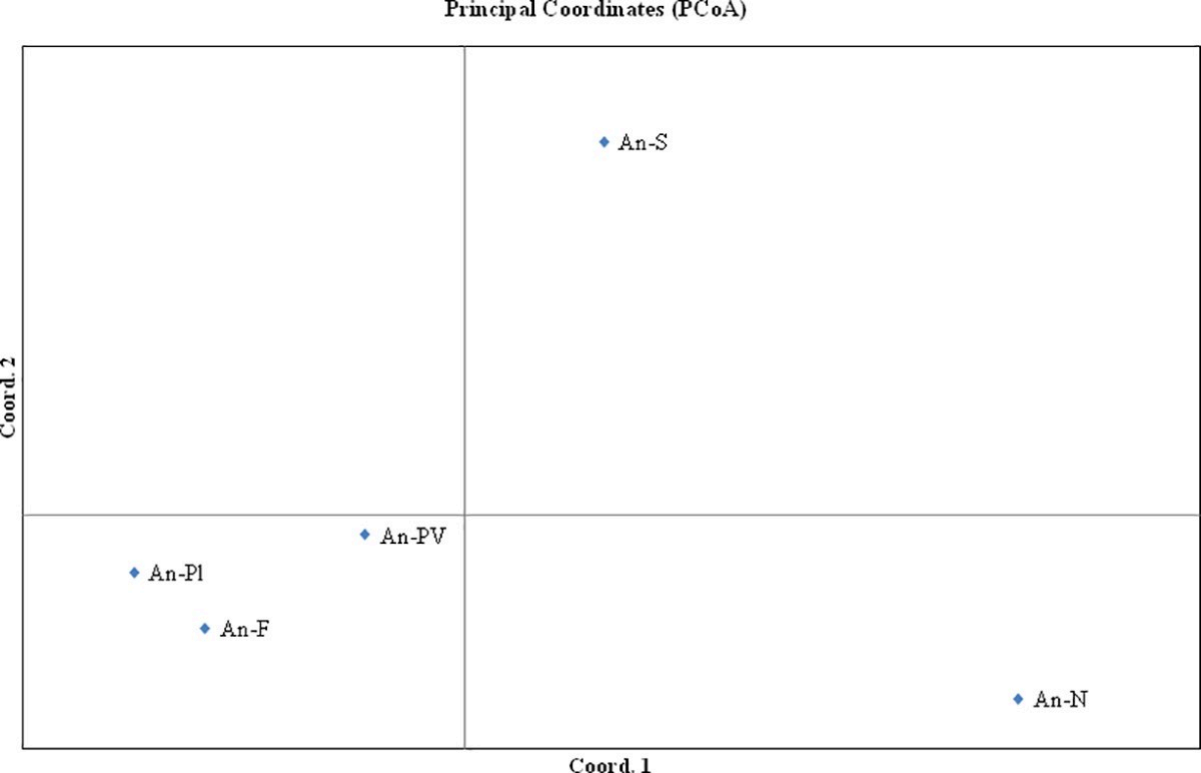
Distribution of variates in a two-dimensional Principal Coordinates Analysis (PCoA) based on corrected F_ST_ values for *Asperula naufraga* subpopulations.

The ΔK statistic in the STRUCTURE analysis presented a maximum peak at K = 3 (Fig 3 and S1 Fig), suggesting that three different genetic clusters exist within the whole population of *A*. *naufraga*. All An-N individuals with some from An-PV an An-F and one individual from An-Pl (slightly decreased *q*-values in the case of An-F individuals) are grouped into one basic cluster, while the remaining individuals are assigned to two different genetic clusters. The major mode (presented in Fig 3) for K = 3 generated by CLUMPAK was supported by 20 out of 20 STRUCTURE runs. All analyses, PCoA based on Nei’s genetic distance, as well as the PCoA based on the corrected *F*st values, are concordant, concluding that subpopulations AN-F and AN-Pl are closely related, while subpopulations An-S and An-N, are more distant. The latter is clearly differentiated from the others, as shown also by the STRUCTURE analysis (Figs 3 and 4).

The estimated *Ne* using the 0.4 *Ne*/*Nc* and 0.1 *Ne*/*Nc* ratio was 52 and 13 individuals, respectively. *Ne* values under 50 individuals indicate that the population’s growth and persistence is likely threatened by inbreeding depression (fixation of deleterious alleles) and loss of alleles [58].

### Population viability analysis

**Fig 5:**
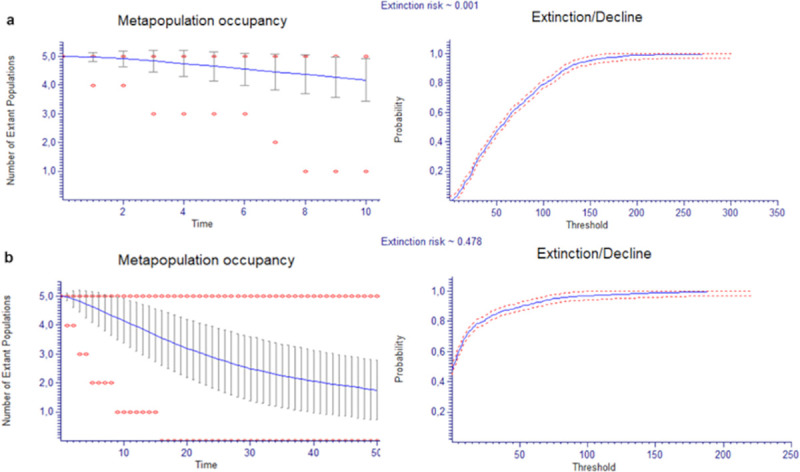
**Population Viability Analysis of *Asperula naufraga* a) in the next 10 and b) in the next 50 years.** The average (line), ±1 standard deviation and minimum and maximum (dots) numbers of the population of *A*. *naufraga* are shown.

PVA in *A*. *naufraga* using the total number of mature individuals was projected for the next 10 and 50 years (Fig 5). Total population seems to have the trend to slightly decrease in the next 10 years (projected extinction risk: 0.1%). During the next 50 years, species extinction risk increases to 47.8%. There is a high possibility for subpopulations An-PV (85.8%), An-S (76.3%) and An-F (66.6%) to go extinct in the next 50 years (S2 Fig).

### Threats

The direct threats recognized for *A*. *naufraga* were: (a) the development of recreational areas (threat code 1.3) and the recreational activities (threat code: 6.1) such as hiking, rock-climbing and the construction of secondary roads (threat code: 4.1) at location An-N; (b) gathering of plants (threat code: 5.2) mainly near the hiking trails at location An-N; (c) increase in frequency of human induced fires at locations An-N and An-PV (threat code: 7.1.1); (d) threats from catastrophic geological events, and more specifically from earthquakes (threat code: 10.2), which result in the destruction of the species and its habitat at locations An-Pl, An-F and An-PV; (e) ranching, i.e. domestic or semi-domesticated animals allowed to roam in the wild (threat code: 2.3.1) at location An-S and An-F.

### Conservation status assessment

Based on monitoring data, *A*. *naufraga* is classified as Critically Endangered (CR) (S3 Fig) according to B1ab(i,ii,iii,v)c(i,ii,iv) and B2ab(i,ii,iii,v)c(i,ii,iv) criteria [41]. More specifically, criterion B1ab(i,ii,iii,v)c(i,ii,iv) refers to the EOO, which is less than 100 km^2^, related to severely fragmented subpopulations, continuing decline observed, estimated, inferred or projected in: EOO, AOO, area-extent-quality of habitat, number of mature individuals, and extreme fluctuations in: EOO, AOO and number of mature individuals. Criterion B2ab(i,ii,iii,v)c(i,ii,iv) refers to AOO, which is less than 10 km^2^, related to severely fragmented subpopulations, continuing decline observed, estimated, inferred or projected in: EOO, AOO, area-extent-quality of habitat, number of mature individuals, and extreme fluctuations in: EOO, AOO and number of mature individuals.

## Discussion

### Population dynamics

Plant population monitoring programs are especially scarce because they are time- and resource- consuming, and are therefore restricted to a few threatened species [59]. Thus, our knowledge about the conservation and biology of most plant species remains remarkably poor. This is even more evident in the case of obligate chasmophytes due to their difficult access, despite the high number of rare and threatened endemics favoring rocky habitats [60]. In this study, all extant subpopulations of *A*. *naufraga*, an emblematic endemic chasmophyte of Zakynthos island, were monitored for five years. The presence of five small and remote subpopulations along about 30 km in the western coastal cliff system of Zakynthos, indicates a long colonization and extinction history. Habitat traits, i.e. the rocky calcareous substrate and open sites with low vegetation cover, were similar in all subpopulations studied. The EOO and the AOO of *A*. *naufraga* were stable during the monitoring period. The local EOO, however, which expresses the true extent of the species in nature, was decreased in subpopulations An-N and An-F due to anthropogenic activities/pressures (e.g. the construction of secondary roads and of hiking trails, exploitation of land for livestock, etc.) and vegetation succession (mainly due to regeneration of *Pinus halepensis* Mill).

The few existing studies on population dynamics of chasmophytes suggest unusually stable population sizes and high local population persistence [11, 61–63]. Interestingly, the population size of *A*. *naufraga* markedly fluctuated over the monitoring period. These annual fluctuations were not only caused by local changes in the habitat or threats/pressures. This becomes clear if we consider that the fluctuation pattern was common to all subpopulations. According to our results, *A*. *naufraga* is a perennial species with a rather short life span. A strong correlation between life span of perennial herbs and population stability has been evidenced [64], while the population size for species with rather short life spans is subject to higher annual variation than for species with longer life spans. This pattern has been linked to higher temporal variability in fecundity than in survival [64, 65], which has also been confirmed in our study for *A*. *naufraga*. Interestingly, maximum fecundity coincided with minimum survival rate of mature individuals during the monitoring period. Moreover, small populations can exhibit strong annual fluctuations in their size due to stochastic forces (e.g. environmental stochasticity) [66]. Environmental changes constrain the survival of single plants and start the progress of consecutive decline of individuals within the population of chasmophytic taxa [67]. The fluctuations in the number of mature individuals of *A*. *naufraga* may be related to climatic conditions (mean temperature and precipitation), assuming that similar environmental factors affect neighboring subpopulations, while the effect of precipitation and mean temperature on population dynamics of perennial herbs has been demonstrated in several studies [e.g. 68, 69]. Certainly, we would need additional data from a longer monitoring period for a statistically robust estimation of the abiotic factors effect on the population dynamics of *A*. *naufraga*. Seedling establishment is especially difficult in vertical cliffs; survival of existing individuals is far more important than the establishment of new plants [14], while high survival rates of mature individuals are vital to the maintenance of chasmophytic populations [14, 64].Highest fecundity, recorded in the year with the minimum number and survival rate of mature individuals (2016), seems to follow the typical perennial plants pattern, that allocate resources between seed production and investment in structures which increase the survival probability and facilitate growth the following year [70].

Flowering phenology is a critical life-history trait that strongly influences reproductive success [71]. The duration of the flowering period of *A*. *naufraga* fluctuates in response to variation in climatic factors (i.e. mean annual temperature and precipitation), while there is a strong positive correlation between the duration of flowering period and RRS. Extended flowering is likely to assure plant reproduction. Individual plants flowering for long periods have several advantages related to higher reproductive success, higher outcrossing rates and more time for seed maturation [72]. For insect-pollinated plants, negative effects of low pollinator visitation can be mitigated by elongating flowering period and floral longevity, which increase the probability of pollinator visitation [73]. In the case of *A*. *naufraga*, the elongated flowering period leads to the production of a larger mean number of seeds, which ultimately leads to higher reproductive success.

Monitoring data collected during this study were used to re-evaluate the conservation status of *Asperula naufraga* according to IUCN categories and criteria [41], using Ramas Red List Professional software [39]. According to our results, *A*. *naufraga* should move from the “Endangered” (EN) to the “Critically Endangered” (CR) category. PVA results indicate that three of the five subpopulations of the species, namely An-PV, An-F and An-S, might go extinct within the next 50 years, while the extinction risk for the whole population is 47.8%.

### Genetic diversity and structure

In this study, three microsatellite loci were used in a preliminary attempt to gather genetic information about the extremely rare and threatened *A*. *naufraga*. The number of alleles revealed for the SSR loci studied, was sufficient to acquire a first rough estimation of genetic variation, characterize the subpopulations, and describe population structure.

Regarding the diversity parameters within *A*. *naufraga*, the Shannon’s information index (*I*) was low, following the same trend as *H*o and u*H*e. Generally, *H*o values are lower than u*H*e values, which may indicate non-random mating [74]. However, we also need to consider that our allele scoring procedure based on the observation of PCR products on agarose gel, may make the scoring prone to allele dropout in addition to the potential occurrence of null alleles. Gene diversity is a measure of genetic variability accounting for allelic richness and evenness in a randomly mating population. *H*o computed on the SSR loci studied, was lower than expected for endemic plant populations. On the other hand, u*H*e values of *A*. *naufraga* are similar to u*H*e values of short lived perennial plants as reported by Nybom [74].

The mean *H*o among all subpopulations was similar to that reported by Gargiulo et al. [29, 32] for *Asperula crassifolia* populations, although in our case u*H*e is higher. Genetic diversity maintained within a species is considered to be a result of both historical events and recent evolutionary processes. Low heterozygosity estimates may be caused by the effect of small population size, genetic drift, high selection pressure in closed population, inbreeding, and restricted gene flow, which eventually contribute to a reduction in genetic diversity [75, 76].

The positive values of fixation index (*F*) for the loci studied indicate a high number of homozygotes. The same also stands for *F*_*IS*_ within subpopulations of *A*. *naufraga* and *F*_*IT*_, which accounts for the entire population. Positive *F*_*IS*_ values may also be recorded in outcrossing populations under certain circumstances. If we exclude the presence of clonal individuals, the causes of such values of *F* indices may be the occurrence of null alleles, non-random mating (which is also verified by the fact that almost in all cases the *A*. *naufraga* subpopulations deviated significantly from Hardy-Weinberg equilibrium), allele dropout, selection and the restricted gene flow (*Nm* = 1.282), especially for the An-N subpopulation. Although we have not specifically tested our data for the occurrence of a recent bottleneck, the low genetic diversity and the relatively high fixation index may suggest that subpopulations of *A*. *naufraga* have suffered from a reduction in their size. In AMOVA, the mean *F*_*ST*_ value is significantly lower than the values reported for narrow/endemic plant populations by Nybom [74], possibly affected by the geographic proximity of subpopulations, also given the small size of Zakynthos island. The *F*_*ST*_ calculated in our study indicates a moderate genetic differentiation, significantly lower than that observed (mean *F*_*ST*_ = 0.293) between the three populations of *A*. *crassifolia* [29, 32]. This is possibly due to differences in their evolutionary history and the large geographic distance and existing barriers among *A*. *crassifolia* populations compared to those of *Α*. *naufraga*.

The mean value of *Nm* was estimated to be 1.282 individuals per generation. However, between subpopulations An-Pl and An-F, *Nm* was much higher (16.840) indicating extensive gene flow, which is also in accordance with the low value for Nei’s D (0.03) between these two subpopulations, as well as their geographical proximity. Interestingly, the individuals from these two subpopulations belong to the same genetic cluster as deduced by the STRUCTURE analysis and they are positioned next to each other in PCoA. There is also increased gene flow between subpopulations An-Pl and An-PV and An-F and An-PV (4.359 and 4.242, respectively). In all other cases, gene flow ranged between 0.893 and 1.686. According to Couvet [77] one migrant per generation may not be sufficient to guarantee long-term survival of small populations. In fact, even 5–20 migrants per generation may not prevent the loss of genetic diversity within and differentiation between populations [78]. Notably, the lowest values of gene flow were observed between An-N and subpopulations An-F and An-Pl. This is also in agreement with the results obtained by the STRUCTURE analysis where An-N is classified in a unique genetic cluster being also the most homogeneous population and the PCoA where An-N is highlighted as the most genetically remote.

Census of the population size facilitate the estimation of *Ne*, which is in line with the results of the Conservation Status Assessment and should be considered as additional information to assessments as suggested by Garner et al. [55].

## Conclusions and conservation remarks

As (census) population size, reproductive aspects and genetic structure of *A*. *naufraga* were unknown, the results of the present study provided relevant new knowledge, crucial for species conservation. Geographical distribution and population size of *A*. *naufraga* are constrained by the availability of suitable habitat and recruitment limitations. Our results indicate that stable population size is not a common feature shared by all perennial obligate chasmophytes. Cliff systems are usually stable habitats, but cliff plant populations may be subject to remarkable fluctuations even in short time scales, as indicated by our monitoring results. The population of *A*. *naufraga* proved to be dominated by adult and senescent individuals, suggesting low recruitment. Survival ability of mature individuals counterbalance recruitment limitations and enhances the survival of the species in harsh environments like cliffs [64, 79, 80], where the opportunities for the establishment of new individuals are scarce. Moreover, the small population size is connected with low genetic diversity and reduced fitness [81], which could impact the species’ ability to adapt to environmental change. Although the extensive cliff systems of western Zakynthos have been thoroughly investigated, the presence of additional small subpopulations growing in inaccessible sites cannot be ruled out. It is improbable, however, that their presence would be sufficient to counterbalance the negative effects of genetic drift and inbreeding.

Population data indicates an increased extinction risk due to the small population size combined with large fluctuations, low recruitment, low genetic diversity and inbreeding. The same is also evidenced by the estimated *Ne*. A pragmatic in situ conservation approach for *A*. *naufraga* should give priority to minimize/eliminate the impacts of the threats it faces. In particular, according to field observations and the results of PVA and genetic analyses, the subpopulations at the locations Faros Keriou, Sxiza and Navagio should be prioritized for conservation measures. The ownership status at the locations of subpopulations An-F, An-S and An-N constitutes a challenge for their conservation. Subpopulations An-F and An-S are partly located in areas that are privately owned, and are also used for animal keeping. Grazing control in order to reduce trampling pressure from herbivores in subpopulations An-F and An-S is required. In addition, informing the local authorities and the community is deemed necessary at the location Navagio, a famous recreational area where accidental trampling and gathering of plants have been observed. Moreover, *ex situ* conservation of the species in seed banks can offer insurance against extinction by providing a source population for future reintroduction or reinforcement of wild population. Seed banking is a necessary and cost-effective complement to in situ conservation of wild plants, and it provides a vital source of material to assist in ecological restoration of damaged and degraded habitats [82]. Genetic analysis indicates that seeds should be collected from all extant subpopulations, with priority to subpopulation An-N, which was highlighted as the most genetically remote.

## Supporting information

S1 Fig Mean of estimated natural log probability (Ln(K) ± s.d.) of STRUCTURE runs of *Asperula naufraga* samples with cluster value (K) ranging from 1–5. (TIF)

S2 Fig Population viability analysis of *Asperula naufraga* subpopulations in the next 50 years. A) Subpopulation An-PV, B) subpopulation An-S, C) subpopulation An-F. The average (line), ±1 standard deviation and minimum and maximum (dots) numbers of the subpopulations of *A*. *naufraga* are shown.
(TIF)

S3 Fig Assessment of conservation status of *Asperula naufraga* using RAMAS Red List Pro software package. (TIF)

S1 Table Allele frequencies at three microsatellite loci in five subpopulations of *A*. *naufraga*. (DOCX)

S2 Table Pairwise population matrix of Nei genetic distance and *F*_ST_ values with and without ENA correction. (DOCX)
