# Supplementary figures and images for: Conservation biology of threatened Mediterranean chasmophytes: The case of Asperula naufraga endemic to Zakynthos island (Ionian islands, Greece)
Source: PLoS One. 2021 Feb 19;16(2):e0246706. doi: 10.1371/journal.pone.0246706 (PMC7894959; doi:10.1371/journal.pone.0246706)

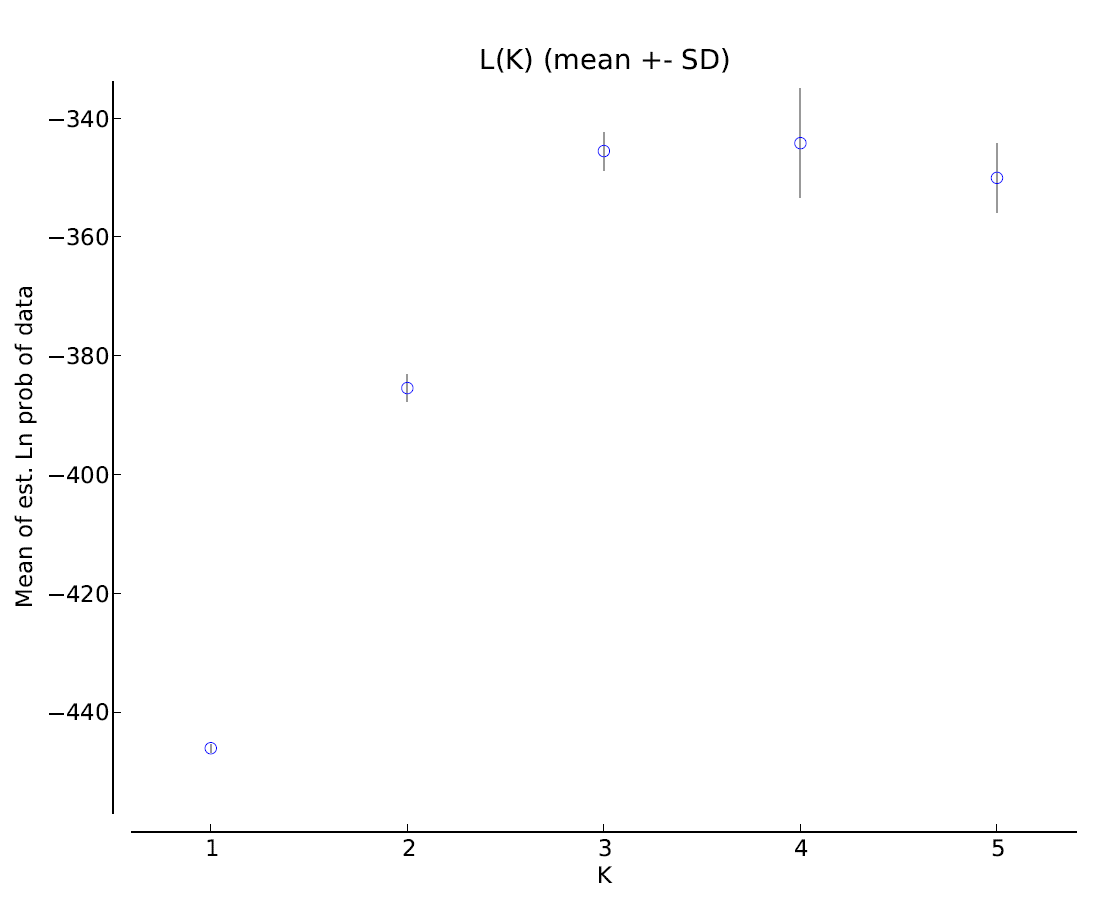

Supplement: S1 Fig — (TIF) [file pone.0246706.s001.tif]

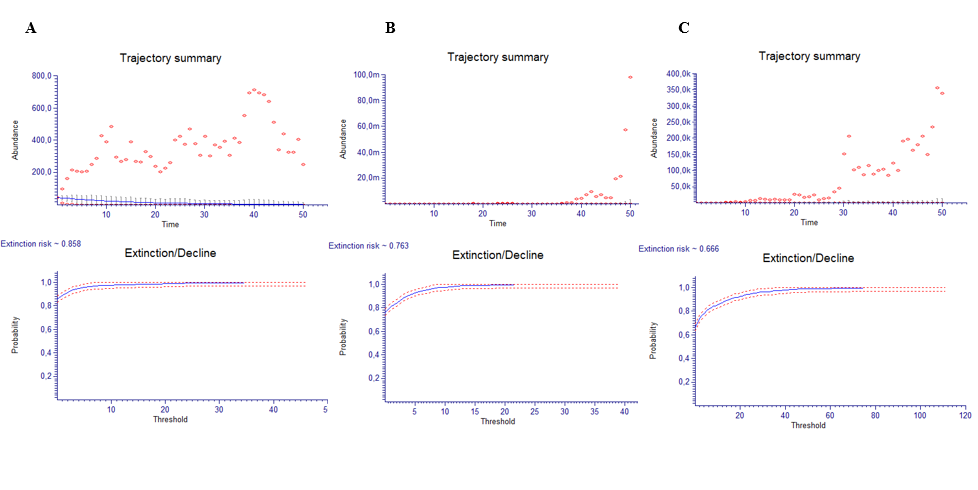

Supplement: S2 Fig — A) Subpopulation An-PV, B) subpopulation An-S, C) subpopulation An-F. The average (line), ±1 standard deviation and minimum and maximum (dots) numbers of the subpopulations of A. naufraga are shown. (TIF) [file pone.0246706.s002.tif]

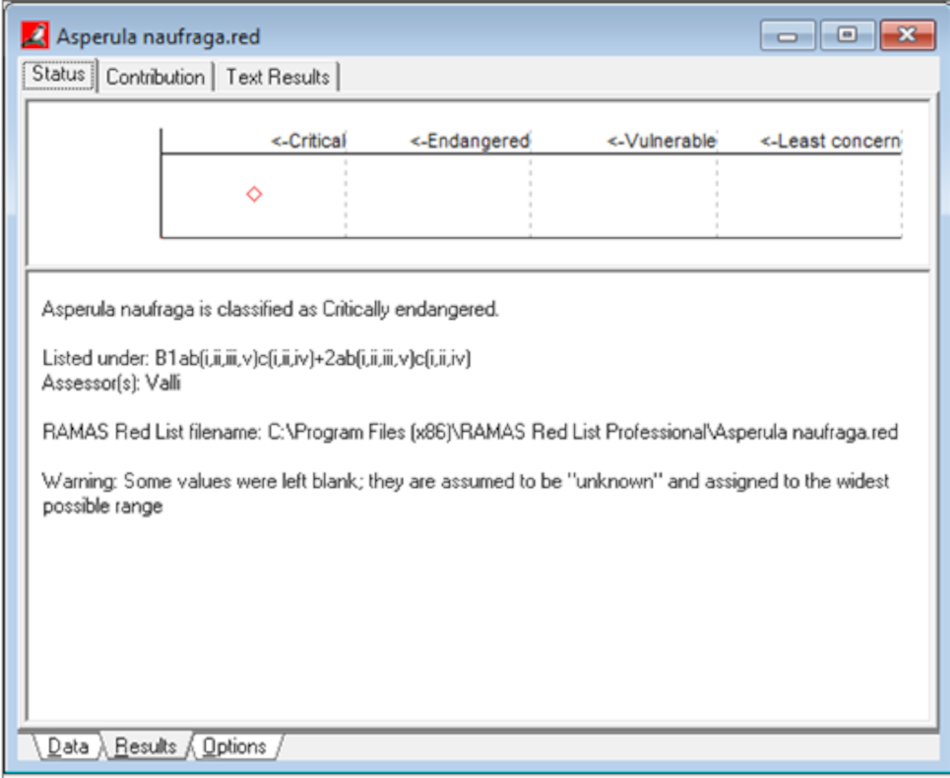

Supplement: S3 Fig — (TIF) [file pone.0246706.s003.tif]
