# Supplementary material for: Conservation biology of threatened Mediterranean chasmophytes: The case of Asperula naufraga endemic to Zakynthos island (Ionian islands, Greece)
Source: PLoS One. 2021 Feb 19;16(2):e0246706. doi: 10.1371/journal.pone.0246706 (PMC7894959; doi:10.1371/journal.pone.0246706)
Supplement: S1 Table — (DOCX) [file pone.0246706.s004.docx]

S1 Table. Allele frequencies at three microsatellite loci in five subpopulations of *A. naufraga.*

| Subpopulation |  | **An-Pl** | **An-F** | **An-PV** | **An-N** | **An-S** |
| --- | --- | --- | --- | --- | --- | --- |
| Locus | Allele |  |  |  |  |  |
| **GA_50A** | 165* | - | - | 0.294 | - | - |
|  | 175 | 0.846 | 0.867 | 0.588 | 0.308 | 0.400 |
|  | 185 | 0.077 | 0.133 | 0.118 | 0.692 | 0.600 |
|  | 201* | 0.077 | - | - | - | - |
| **GA_30C** | 190 | 0.038 | - | 0.324 | - | 0.400 |
|  | 210 | 0.500 | 0.400 | 0.412 | 0.500 | 0.600 |
|  | 216 | 0.231 | 0.200 | 0.088 | 0.154 | - |
|  | 220 | 0.231 | 0.400 | 0.176 | 0.346 | - |
| **GA_1** | 150 | 0.038 | - | 0.147 | 0.577 | - |
|  | 156 | 0.654 | 0,533 | 0.471 | - | 0.200 |
|  | 160 | 0.038 | 0.200 | 0.206 | 0.423 | - |
|  | 176 | 0.269 | 0.267 | 0.176 | - | 0.800 |

* Private alleles to specific subpopulations, as indicated
