# Supplementary material for: Conservation biology of threatened Mediterranean chasmophytes: The case of Asperula naufraga endemic to Zakynthos island (Ionian islands, Greece)
Source: PLoS One. 2021 Feb 19;16(2):e0246706. doi: 10.1371/journal.pone.0246706 (PMC7894959; doi:10.1371/journal.pone.0246706)
Supplement: S2 Table — (DOCX) [file pone.0246706.s005.docx]

| Pairwise Population Matrix of Nei Genetic Distance | | | | |  | | Pairwise *F*_ST_ values using the ENA correction | | | | | | | Pairwise *F*_ST_ values without the ENA correction | | | | |
| --- | --- | --- | --- | --- | --- | --- | --- | --- | --- | --- | --- | --- | --- | --- | --- | --- | --- | --- |
|  | An-PL | An-F | An-PV | An-N | | An-S | | An-PL | An-F | An-PV | An-N | An-S | An-PL | | An-F | An-PV | An-N | An-S |
| An-PL | * |  |  |  | |  | | * |  |  |  |  | * | |  |  |  |  |
| An-F | 0.030 | * |  |  | |  | | -0.012 | * |  |  |  | -0.024 | | * |  |  |  |
| An-PV | 0.124 | 0.136 | * |  | |  | | 0.057 | 0.034 | * |  |  | 0.045 | | 0.047 | * |  |  |
| An-N | 0.759 | 0.614 | 0.568 | * | |  | | 0.310 | 0.277 | 0.165 | * |  | 0.323 | | 0.276 | 0.203 | * |  |
| An-S | 0.458 | 0.495 | 0.394 | 0.643 | | * | | 0.210 | 0.202 | 0.147 | 0.290 | * | 0.239 | | 0.232 | 0.128 | 0.280 | * |

S2 Table. Pairwise Population Matrix of Nei genetic distance and FST values with and without ENA correction.
